# 7-Epiclusianone, a Benzophenone Extracted from *Garcinia brasiliensis* (*Clusiaceae*), Induces Cell Cycle Arrest in G1/S Transition in A549 Cells

**DOI:** 10.3390/molecules200712804

**Published:** 2015-07-15

**Authors:** Marisa Ionta, Guilherme A. Ferreira-Silva, Evandro L. Niero, Éderson D’Martin Costa, Adam A. Martens, Welton Rosa, Marisi G. Soares, Gláucia M. Machado-Santelli, João Henrique G. Lago, Marcelo H. Santos

**Affiliations:** 1 Institute of Biomedical Sciences, Federal University of Alfenas, 37130-000 Alfenas, Brazil; E-Mail: alfer_gui@hotmail.com; 2 Department of Cell and Developmental Biology, Institute of Biomedical Sciences, University of Sao Paulo, 05508-000 São Paulo, Brazil; E-Mails: eloniero@yahoo.com (E.L.N.); adam.arai@gmail.com (A.A.M.); glaucia.usp@gmail.com (G.M.M.-S.); 3 Institute of Chemistry, Federal University of Alfenas, 37130-000 Alfenas, Brazil; E-Mails: edm_quimica@yahoo.com.br (E.D.C.); welton_rosa@hotmail.com (W.R.); marisigs@gmail.com (M.G.S.); 4 Institute of Environmental, Chemical and Pharmaceutical Sciences, Federal University of Sao Paulo, 09920-000 Diadema, Brazil; E-Mail: joao.lago@unifesp.br; 5 Institute of Chemistry, Federal University of Viçosa, 36570-900 Viçosa, Brazil; E-Mail: marcelo_hs@yahoo.com.br

**Keywords:** 7-epiclusianone, *Garcinia brasiliensis*, antiproliferative activity, lung cancer, cell cycle arrest, cell death

## Abstract

Lung cancer is the leading cause of cancer deaths in the world. Disease stage is the most relevant factor influencing mortality. Unfortunately, most patients are still diagnosed at an advanced stage and their five-year survival rate is only 4%. Thus, it is relevant to identify novel drugs that can improve the treatment options for lung cancer. Natural products have been an important source for the discovery of new compounds with pharmacological potential including antineoplastic agents. We have previously isolated a prenylated benzophenone (7-epiclusianone) from *Garcinia brasiliensis* (*Clusiaceae*) that has several biological properties including antiproliferative activity against cancer cell lines. In continuation with our studies, the present work aimed to investigate the mechanisms involved with antiproliferative activity of 7-epiclusianone in A549 cells. Our data showed that 7-epiclusianone reduced the viability of A549 cells in a concentration-dependent manner (IC_50_ of 16.13 ± 1.12 μM). Cells were arrested in G1/S transition and apoptosis was induced. In addition, we observed morphological changes with cytoskeleton disorganization in consequence of the treatment. Taken together, the results showed that cell cycle arrest in G1/S transition is the main mechanism involved with antiproliferative activity of 7-epiclusianone. Our results are promising and open up the prospect of using this compound in further anticancer *in vivo* studies.

## 1. Introduction

Lung cancer is the leading cause of cancer deaths in the world [1] and can be divided in two main forms: non-small cell lung cancer (NSCLC) and small-cell lung cancer (SCLC), which represent, respectively, 85% and 15% of all diagnosed lung cancers [2]. NSCLC is composed of three histological subtypes: squamous cell carcinomas (SSCs), lung adenocarcinoma and large-cell lung carcinoma (LCLC). Among these subtypes, adenocarcinoma is the most prevalent [3]. Lung cancer cell populations are usually heterogeneous but *in vitro* models may help us to understand molecular events responsible for modulating cell behavior in response to different stimulus [4].

Natural products have been an important source for the discovery of new antineoplasic therapeutic agents [5], and plants are especially rich in such compounds due to their significant chemodiversity. For example, *Garcinia brasiliensis* (*Clusiaceae*) produces several metabolites [6] including benzophenones such as 7-epiclusianone, which displayed a wide spectrum of biological activities [7,8,9,10,11,12] including antiproliferative potential against a panel of tumor cell lines [13]. Following up with our previous studies, here we investigated the possible mechanisms involved in the antitumor activity of 7-epiclusianone. Thus, a cell line derived from human lung cancer (A549) was used as a model to investigate the influence of 7-epiclusianone on cell cycle progression, apoptosis induction and cytoskeleton network. Our data open up the prospect of using 7-epiclusianone as a scaffold for the design of novel and selective drug candidates for further *in vivo* studies and it is a promising tool for the development of new therapeutic agents for lung cancer treatment.

## 2. Results and Discussion

### 2.1. Antiproliferative and Cytotoxic Activity

**Figure 1:**
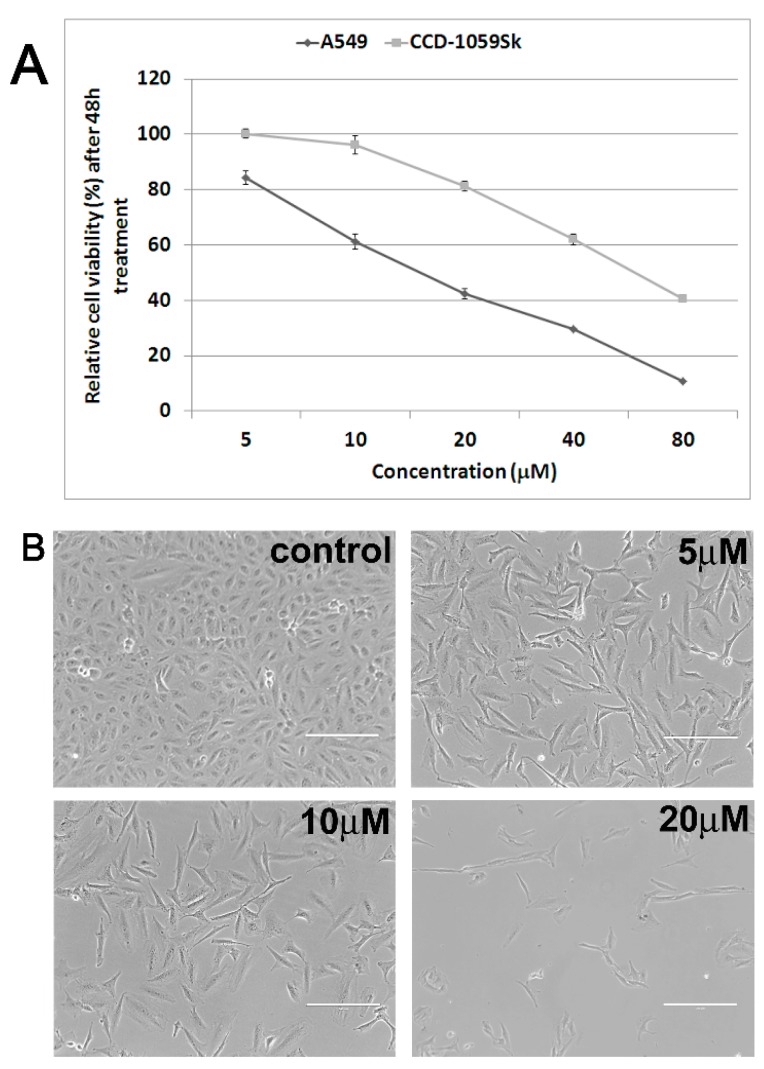
(**A**) Cell viability profile of A549 and CCD-1059Sk cells after treatment with 7-epiclusianone for 48 h; (**B**) Phase contrast microscopy images showing morphological aspect of A549 cells. 7-epiclusianone treatment clearly affected cell density in a concentration-dependent manner and induced cell morphology changes. Scale bars: 200 µm.

7-Epiclusianone was isolated from ethanolic extract from fruit epicarps of *G. brasiliensis* by successive chromatographic steps and characterized by NMR and MS spectral analysis. Different dilutions of this compound were used to treat A549 lung cancer cells, and we found antiproliferative and pro-apoptotic effects in a concentration-dependent manner. After 48 h, the treatment caused a drastic reduction in cell viability (Figure 1A) indicating an IC_50_ value of 16.13 ± 1.12 μM. The antiproliferative activity of 7-epiclusianone was superior to cisplatin, a widely used chemotherapeutic agent (IC_50_ = 21.71 ± 1.17 μM). We also investigated the cytotoxic activity of 7-epiclusianone in normal fibroblasts (CCD-1059Sk) and the IC_50_ value was 3.6-fold higher when compared to A549 cells. It is important to note that the proliferation rate of CCD-1059Sk cells is lower than A549 cells (data not shown) and therefore the difference observed between the IC_50_ values could be associated with the different proliferative behavior of these cells. Despite the fact that a remarkable antiproliferative activity of 7-epiclusianone on PC03 (kidney), 786-0 (prostate), UACC (melanoma), and OVCAR (ovarian) tumor cell lines had been previously reported [13], the molecular mechanisms involved remained unclear.

**Figure 2:**
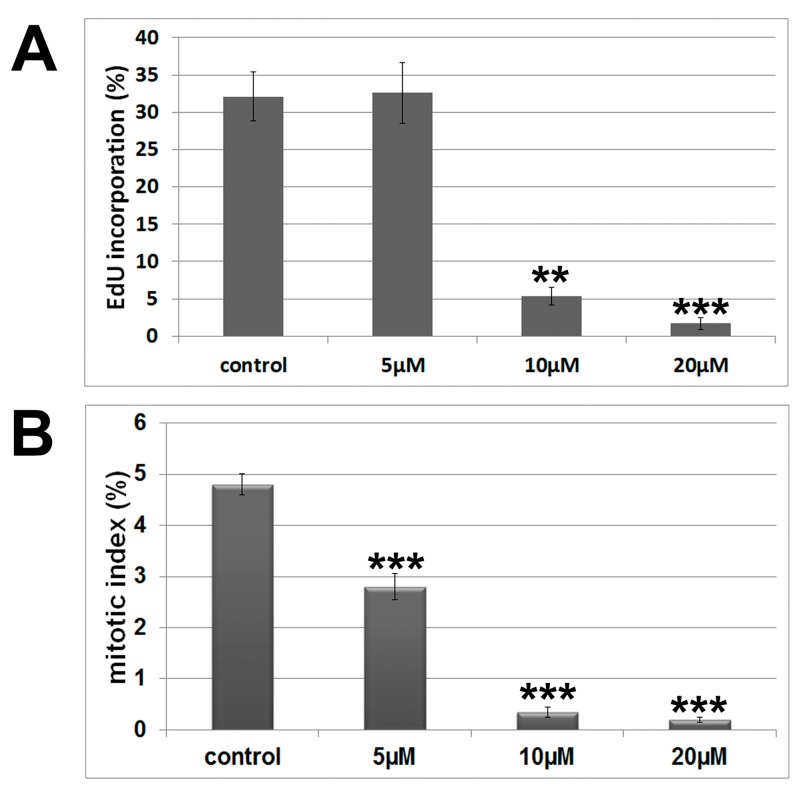
(**A**) Histograms showing the EdU incorporation rate after treatment with different concentrations of 7-epiclusianone for 48 h; (**B**) Mitotic indices determined from cytological preparations that were immunostained for tubulin, with propidium iodide-stained nuclei. Significant differences to control results were determined using ANOVA followed by Tukey’s post-test. ** *p* < 0.01 and *** *p* < 0.001.

Images obtained by phase contrast microscopy (Figure 1B) evidenced reduction in cellular density in a concentration-dependent manner. Besides, treated cells changed their typical epithelial-like morphology to elongated or fusiform shapes. Flow cytometry analysis showed a significant increase (*p* ˂ 0.05) in the G1 population after treatment (control 62.10%, 5 μM 73.83% and 10 μM 75.20%) with a concomitant decrease (*p* ˂ 0.05) in the S population (control 19.77%, 5 μM 9.84% and 10 μM 5.53%) (Table 1). These results suggest that 7-epiclusianone induces cell cycle arrest in G1/S transition. To confirm this data, DNA synthesis was analyzed by EdU assay, a specific method to evidence cell population in S-phase [14]. EdU assay results corroborated our earlier observations, *i.e.*, there was a significant reduction in the frequency of S-phase cells (Figure 2A). Therefore, we demonstrated that the antiproliferative activity of 7-epiclusianone on A549 cells is, at least in part, associated with its capability of inducing cell cycle arrest in G1/S transition. Up to date, there were no data in the literature reporting the negative control of 7-epiclusianone on cell cycle progression of cells derived from solid tumors. It has been reported that other polyisoprenylated benzophenones such as guttiferone H, guttiferone E, and xanthochymol, isolated from *G. santhochymus* fruits, induced cell cycle arrest in colon cancer cell lines [15]. Cell cycle arrest in G1/S transition has also been described in PaCa (pancreatic cancer cells) after treatment with garcinol, a benzophenone isolated from *G. indica* [16].

According to flow cytometry analysis, no significant alteration was observed in G2/M population when treated cultures at 10 μM 7-epiclusianone (17.48%) were compared to control groups (17.23%). However, there was a significant (*p* < 0.05) reduction in G2/M population after treatment with 5 μM 7-epiclusianone (15.60%). Interestingly, the mitotic indices were significantly lower (*p* < 0.001) in all treated groups in relation to controls (Figure 2B).

**Table 1:** Cell cycle analysis after 48 h of treatment with 7-epiclusianone.

Cell Cycle Phases	Control	5 µM	10 µM
**Sub-G1**	0.90 ± 0.14	0.73 ± 0.17	1.79 ± 0.16 ^a^
**G0/G1**	62.10 ± 1.06	73.83 ± 1.97 ^a^	75.20 ± 1.70 ^a^
**S**	19.77 ± 2.34	9.84 ± 0.80 ^a^	5.53 ± 0.43 ^a^
**G2/M**	17.23 ± 1.64	15.60 ± 1.55 ^a^	17.48 ± 1.94

^a^ Significantly different compared to control results (*p* < 0.05). Data were analyzed using ANOVA followed by Tukey’s *post*-test.

G1- and G2-phase arrest usually occurs in response to DNA damage. In general, cells that express wild-type p53 normally exhibit arrest in G1-phase as a consequence of the G1-checkpoint activation, whereas cells that present p53 mutations or deficiency in the P53 signaling pathway present arrest in G2 phase [17,18]. The cells used in the present study (A549) express wild-type p53. Hence, the observed cell cycle arrest in G1/S transition could be a consequence of the P53 pathway activation.

**Figure 3:**
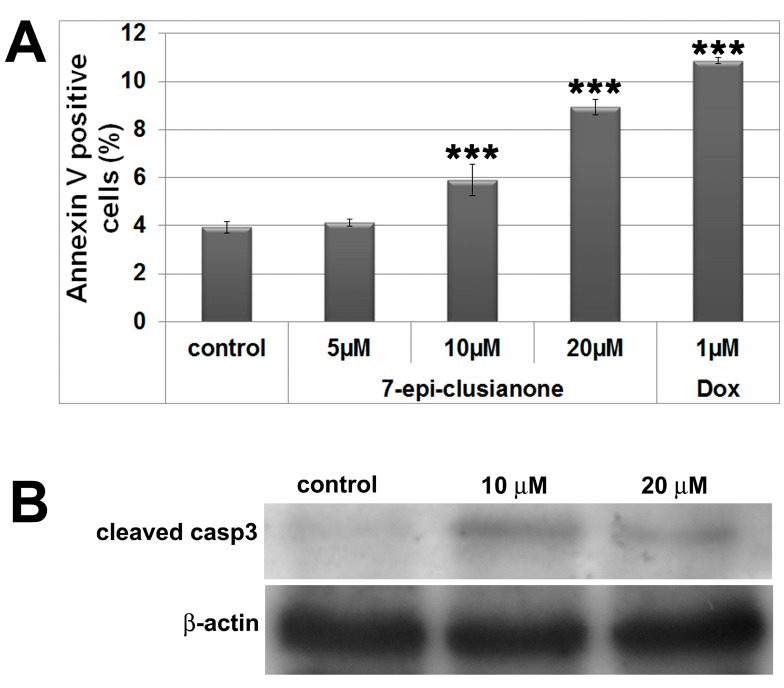
(**A**) Cell death evaluated by Annexin V assay; doxorubicin was used as a positive control; (**B**) Immunoblot for cleaved caspase 3; β-actin was used as loading control. Significant differences compared to control results are indicated. *** *p* < 0.001.

Sub-G1 population was higher (1.79%) in the culture treated with 10 μM 7-epiclusianone when compared to control (0.90%) (*p* < 0.05). Thus, the pro-apoptotic effect of 7-epiclusianone on A549 cells was investigated by an annexin V assay and immunoblot. Our results showed 5.89% and 8.93% of cells positive for annexin V in the samples treated with 10 μM and 20 μM 7-epiclusianone, respectively, compared to 3.93% for the control (Figure 3A). Furthermore, cleaved caspase 3 was detected by immunoblot in treated cells in opposition to control samples (Figure 3B). Therefore, our results demonstrated that 7-epiclusianone induces apoptosis in A549 cells, an effect similar to those reported for leukemia [19] and pancreatic cancer cells [16].

### 2.2. Cytoskeleton and Cell Morphology

**Figure 4:**
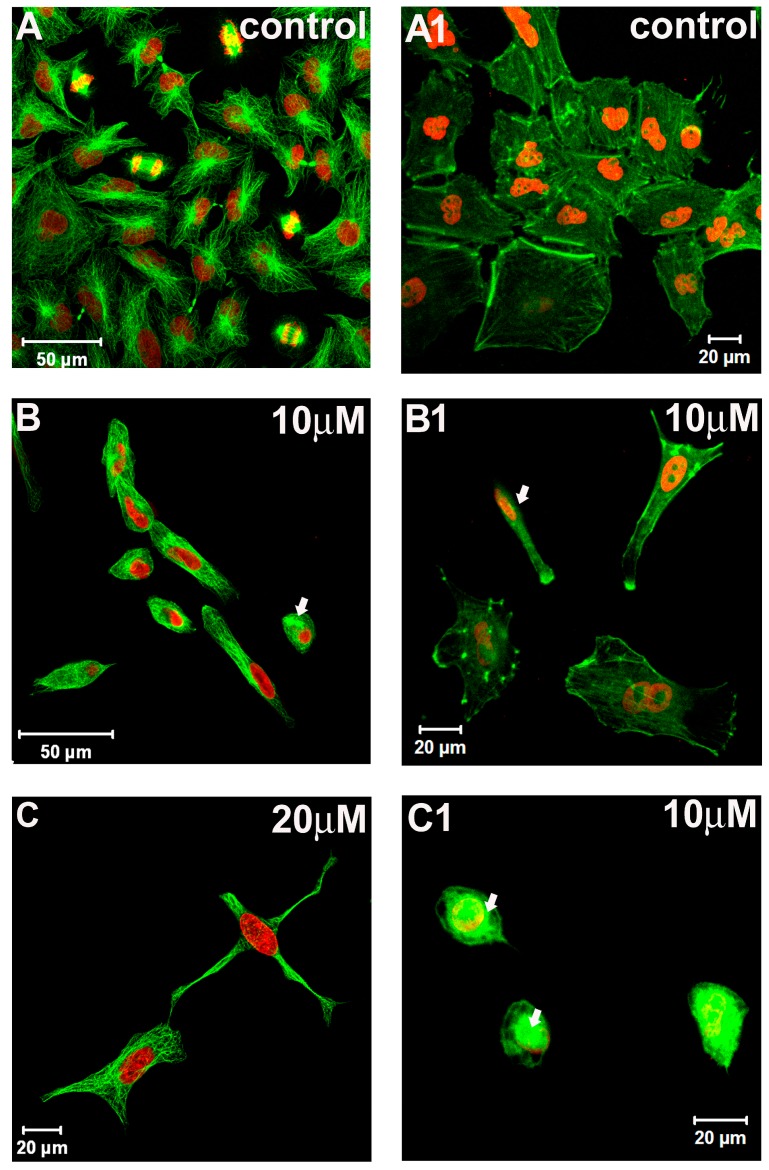
Images obtained by laser-scanning confocal microscopy evidencing microtubule network (**A**–**C**) and microfilament distribution pattern (**A1**–**C1**) in A549 cells in presence or absence of 7-epiclusianone. Nuclei were stained with propidium iodide (red).

We further investigated the effect of 7-epiclusianone on microtubule and actin filaments organization. Images obtained by laser-scanning confocal microscope (LSCM) evidenced the microtubule distribution pattern in control (Figure 4A) and treated cells (Figure 4B,C). The treatment affected the normal distribution pattern of the microtubules since coalesced microtubules were frequently observed in the cytoplasm of treated cells (Figure 4B, arrow). In addition, cells with dendritic-like morphology were also observed (Figure 4C).

Microtubules are the target of drugs commonly used in cancer therapy such as taxol-like compounds and vinca alkaloids [20]. Drugs that affect microtubule dynamics usually induce cell cycle arrest in M phase [21], eventually leading cells to apoptosis. Here, we did not observe cells arrested in M phase, suggesting that the alterations in normal distribution pattern of microtubules is not caused by a direct interaction between 7-epiclusianone and these cytoskeleton elements. Thus, apoptosis induction may not be directly related to microtubules disorganization, as observed with cinnamic acid in melanoma cells [22].

Morphological features of the A549 cells were evaluated in cytological preparations performed for actin filaments. Control cultures showed cells arranged in clusters with polyhedral morphology and actin filaments were evidenced across the cytoplasm forming stress fibers (Figure 4A1). By contrast, neither cell clusters nor actin stress fibers across the cytoplasm were observed in treated cultures (Figure 4B1,C1, and arrow in Figure 4B1, respectively).

Furthermore, we observed small and rounded cells with microfilament network disruption in cultures treated with 10 μM 7-epiclusianone (Figure 4C1, arrow). Actin filaments are associated with cell motility and cell spreading and, therefore, represent an important target for chemotherapeutic drugs [23]. These results demonstrated that 7-epiclusianone was effective in altering the normal networks of both microtubule and microfilaments in A549 cells.

In conclusion, the present work established for the first time that 7-epiclusianone induces cell cycle arrest in G1/S transition and apoptosis in A549 cells. Moreover, the treatment causes microfilament and microtubule disruption. These effects on cytoskeleton elements are an important evidence of the cytotoxic activity of 7-epiclusianone and must be further investigated. We suggest that 7-epiclusianone could be a valuable tool in lung cancer therapy.

## 3. Experimental Section

### 3.1. General Procedures

^1^H- and ^13^C-Nuclear Magnetic Resonance (NMR) spectra were acquired at 300 and 75 MHz, respectively, in a Bruker Avance 300 spectrometer (Bremen, Germany). Samples were dissolved in C_6_D_6_ containing 1% of TMS (Tedia, Rio de Janeiro, Brazil). Low-resolution electron impact mass spectra (LREIMS) were acquired in a HP 5990/5988A mass spectrometer. Silica gel 60 (63–200 μm, Merck, Darmstadt, Germany) and 60 PF_254_ (Merck) were used for adsorption chromatography and for analytical TLC (0.25 mm), respectively. 

### 3.2. Isolation of 7-Epiclusianone from G. Braziliensis

**Figure 5:**
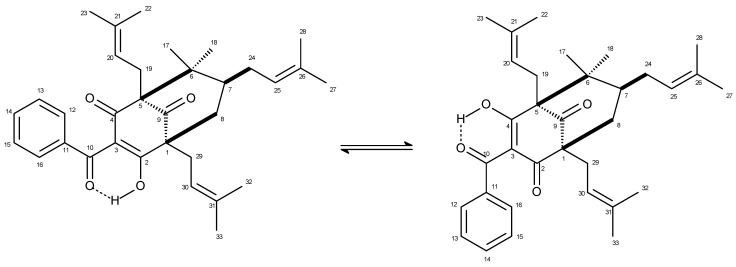
Tautomeric forms of 7-epiclusianone.

Fruits of *G. brasiliensis* were collected in the Campus of Universidade Federal de Viçosa (UFV), Viçosa, Minas Gerais State, Brazil, as previously described [6]. The dried fruit epicarps (500 g) were milled and extracted by maceration using EtOH (3 L) during 24 h. Evaporation of the solvent under reduced pressure afforded the crude EtOH extract, which was chromatographed on SiO_2_ column. Elution with increasing amounts of EtOAc in hexane afforded three groups (A–C). Group B was washed with acetone and the soluble phase, after solvent evaporation, was chromatographed on SiO_2_ column and eluted with increasing amounts of EtOAc in hexane to afford five groups (B1–B5). Two tautomeric forms of 7-epiclusianone (Figure 5) were isolated as crystalline needles from group B4 (1000 mg) after recrystallization using MeOH as solvent [24]. Its structure was defined by analysis of ^1^H- and ^13^C-NMR, as well as LREIMS spectra, and comparison with data reported in the literature [25].

*7-Epiclusianone*. Yellow crystalline needles (MeOH); mp 92–93 °C; LREIMS *m*/*z* (rel. int.) 502 [M]^+^ (5) (corresponding to C_33_H_42_O_4_), 433 (100), 309 (53), 105 (21), 69 (15); ^1^H-NMR (300 MHz, C_6_D_6_) δ/ppm: 7.60 (m, H-12 and H-16), 7.07 (m, H-14), 7.00 (m, H-13 and H-15), 5.69/5.57 (m, H-30), 5.58/5.28 (m, H-20), 4.81/4.79 (m, H-25), 2.89/2.65 (m, H-19), 2.75/2.62 (m, H-29), 2.29/2.18 (dd, *J* = 14 and 1 Hz, H-8a), 2.19 (m, H-24), 2.09/1.86 (dd, *J* = 14 and 7 Hz, H-8b), 1.71/1.70 (s, H-22), 1.71/1.67 (s, H-32 and H-33), 1.60/1.53 (s, H-27),1.56/1.45 (s, H-23), 1.50/1.40 (s, H-28), 1.24 (m, H-7), 1.14/1.07 (s, H-17), 0.86/0.81 (s, H-18). ^13^C-NMR (75 MHz, C_6_D_6_) δ/ppm: 207.9/207.8 (C-9), 198.2/197.8 (C-2), 197.4/196.8 (C-10), 193.5/197.8 (C-4), 137.2/137.3 (C-11), 135.0 (C-21), 134.8 (C-31), 132.9/132.5 (C-14),132.6/132.9 (C-26), 129.6 (C-12 and C-16), 127.8 (C-13 and C-15), 124.8/124.2 (C-25), 120.9/120.6 (C-30), 119.8/119.4 (C-20), 116.5 (C-3), 69.0/66.1 (C-5), 58.9/63.4 (C-1), 48.2/48.9 (C-6), 46.7/46.9 (C-7), 40.6/39.4 (C-8 and C-29), 29.4/29.2 (C-24), 27.2/26.6 (C-19), 27.0 (C-18), 26.2 (C-33), 26.0 (C-23), 25.8 (C-27), 23.0/22.5 (C-17), 18.3 (C-32), 18.2/18.1 (C-22), 17.8 (C-28).

To perform biological assays, 7-epiclusianone was dissolved in DMSO and the stock solution was diluted in fresh medium at appropriate concentrations immediately before use. The final concentration of DMSO in media did not exceed 0.5% (*v*/*v*).

### 3.3. Cell Culture Conditions

A549 cell line (human lung adenocarcinoma epithelial cells) and CCD-1059Sk (human skin fibroblasts) were obtained from Rio de Janeiro cell Bank (BCRJ) (Rio de Janeiro, Brazil). Cell cultures were maintained in DMEM (Dulbecco’s Modified Eagle’s Medium, Sigma, St. Louis, MO, USA/Ham-F12 (Sigma) supplemented with 10% fetal bovine serum (FBS, Cultilab, Sao Paulo, Brazil). Cells were grown in a 37 °C humidified incubator containing 5% CO_2_.

### 3.4. Cell Viability

Cell viability was evaluated by MTS assay. Cells were plated into 96-wells at a density of 5 × 10^3^ cells/well. After attachment (24 h), the cells were treated for 48 h with 7-epiclusianone at different concentrations (5, 10, 20, 40 or 80 μM). The treatment period (48 h) was determined from a pilot study in which the cells were treated for 24, 48 and 72 h with 7-epiclusianone (data not shown). Promega non-radioactive cell proliferation assay was used to determinate cell viability. This assay measures the amount of formazan produced from [3-(4,5-dimethylthiazol-2-yl)-5-(3-carboxymethoxyphenyl)-2-(4-sulfophenyl)-2*H*-tetrazolium, inner salt, MTS] by the dehydrogenase enzymes of metabolically active cells. Thus, the quantity of formazan produced (as measured by absorbance at 490 nm) is directly proportional to the number of living cells. Absorbance values of the treated cells were compared to the absorbance values of untreated cells. Experiments were conducted in triplicate wells and repeated twice. Means ± standard deviations (SD) were calculated. The IC_50_ value was determined by non-linear regression using GraphPad Prism^®^ (GraphPad Software, Inc., San Diego, CA, USA).

### 3.5. Cell Cycle Analysis

To analyze cell cycle progression, cells were treated with 7-epiclusianone for 48 h at 5 μM or 10 μM. The cells were fixed with 75% methanol at 4 °C overnight, then rinsed twice with cold phosphate-buffered saline (PBS). Following, cells were resuspended in dye solution (PBS containing 30 μg/mL propidium iodide (PI) and 3 mg/mL RNAase). DNA was quantified 1 h after staining. The analysis was performed by flow cytometry (Guava easyCyte 8HT, Hayward, CA, USA). The experiments were conducted in triplicate and repeated twice. Data are presented as mean ± SD. 

### 3.6. Annexin V Assay

FITC Annexin V Apoptosis Detection Kit I (BD Pharmigen, San Diego, CA, USA) was used to evaluate phosphatidylserine externalization in apoptotic cells [26]. Samples were treated with 7-epiclusianone at 5, 10 or 20 μM for 48 h. Briefly, cells were collected, rinsed twice with cold PBS, and resuspended (1 × 10^6^ cells/mL) in 1X binding buffer. Then, 5 μL annexin-FITC and 5 μL PI were added. After a 15-min incubation period at room temperature in the dark, cells were analyzed by flow cytometry. The experiments were conducted in triplicate and repeated twice. Data are presented as mean ± SD.

### 3.7. Cytoskeleton Elements: Microtubules and F-Actin

Cells were seeded on coverslips in 35 mm dishes at 7 × 10^4^ cells/plate. On the next day, the medium was replaced with fresh medium containing 10 μM or 20 μM 7-epiclusianone [27]. Next, cells were fixed with 3.7% formaldehyde for 30 min, permeabilized with Triton X-100 (0.5%) for 10 min and incubated with specific primary and secondary antibodies. Then, cells were labeled with phalloidin–FITC and nuclei were stained with propidium iodide (10 mg/mL). After RNAse treatment, coverslips were mounted on histological slides using Vecta-Shield (Vector Laboratories, Burligame, CA, USA). The analyses were performed using a confocal laser-scanning microscope (LSM 510, Zeiss, Oberkochen, Germany), using argon (488 nm) and helium–neon (543 nm) lasers.

### 3.8. Mitotic Index

Mitotic cells were counted from fluorescent cytological preparations stained for microtubules and nuclei; 1000 cells were counted in each sample. The experiments were performed in triplicates. Data are shown as the mean plus SD obtained from three independent experiments.

### 3.9. S-phase Determination Using EdU Assay

Cells were seeded on coverslips in 35 mm dishes at 7 × 10^4^ cells/plate. On the next day, the medium was replaced with fresh medium containing 5, 10 or 20 μM 7-epiclusianone. After 48 h, samples were incubated with fresh DMEM/F12 medium supplemented with 10% FBS containing 10 μM 5-ethynyl-2′-deoxyuridine (EdU, Click-iT TM EdU Imaging Kit—Invitrogen, Carlsbad, CA, USA) for 30 min. Following, cells were fixed with formaldehyde (3.7%) for 30 min and permeabilized with Triton X-100 (0.5%) for 15 min. EdU detection was performed according to manufacturer’s instructions. Cells were treated with RNAse (10 mg/mL) and nuclei were stained with propidium iodide (10 μg/mL). Coverslips were mounted on histological slides using Vecta-Shield (Vector Laboratories). The analysis was performed using a confocal laser-scanning microscope (LSM 510, Zeiss, Oberkochen, Germany).

### 3.10. Immunoblot

Cells were cultured in 75 cm^2^ flasks and then incubated with 7-epiclusianone at 10 or 20 μM for 48 h. After treatment, cells were washed with cold PBSA and scraped into RIPA buffer (150 mM NaCl, 1.0% Nonidet P-40, 0.5% deoxycholate, 0.1% SDS and 50 mM Tris pH 8.0) containing both protease and phosphatase inhibitors (Sigma). Lysates were centrifuged (10,000× *g*) for 10 min at 4 °C. The supernatants were recovered, total proteins were quantified (BCA kit, Pierce Biotechnology Inc., Rockford, IL, USA) and resuspended in Laemmli sample buffer containing 62.5 mM Tris–HCl pH 6.8, 2% SDS, 10% glycerol, 5% 2-mercaptoethanol and 0.001% bromophenol blue. An aliquot of 30 μg protein was separated by SDS–PAGE (12%) and transferred (100 V, 250 mA for 2 h) onto a PVDF membrane (Amersham Bioscience/GE Healthcare, New York, NY, USA), which was blocked by incubation for 1 h at 4 °C with block solution (5% non-fat milk in Tris-buffered saline (TBS) + 0.1% (*v*/*v*) Tween-20). Membrane was probed with cleaved caspase 3 antibody (cell signaling—1:200) overnight at 4 °C. After washing with TBS-tween (0.1%), the membrane was incubated with secondary antibody (anti-rabbit peroxidase-conjugated) for 2 h at room temperature. Immunoreactive bands were visualized with the ECL Western blotting detection Kit (Amersham Biocience/GE Healthcare) according to the manufacturer’s instructions. Anti-β-actin antibody was used as loading control.

### 3.11. Statistical Analysis

Quantitative data are presented as mean ± S.D. (standard deviation). Experiments were conducted in triplicate and ANOVA tests were performed for each, followed by Tukey’s comparison test when relevant. Statistical tests and analyses were performed using GraphPad Prism^®^ (GraphPad Software, Inc., San Diego, CA, USA) software.

## 4. Conclusions 

The present work consistently demonstrated that 7-epiclusianone, a tetraprenylated benzophenone, induces cell cycle arrest in G1/S transition and apoptosis in A549. Our data open up the prospect of using 7-epiclusianone as a scaffold for the design of novel and selective drug candidates for further *in vivo* studies and it is a promising tool for the development of new therapeutic agents for lung cancer treatment.
